# Interstitial Granulomatous Drug Reaction: A Case Report

**DOI:** 10.7759/cureus.21893

**Published:** 2022-02-04

**Authors:** Raghad T Aldibane, Khalid Al Hawsawi

**Affiliations:** 1 Dermatology Department, King Abdulaziz Hospital, Makkah, SAU

**Keywords:** granuloma annulare, reactive granulomatous dermatitis, palisaded neutrophilic granulomatous dermatitis, interstitial granulomatous dermatitis, interstitial granulomatous drug reaction

## Abstract

Interstitial granulomatous drug reaction (IGDR) is a rare inflammatory reaction of the skin with an unknown etiology. Here, we report the case of a 55-year-old female with a history of diabetes mellitus and hypertension who presented with an asymptomatic persistent skin lesion over the left breast for more than one year. Skin examination revealed a single non-scaly, sharply demarcated, erythematous annular patch. Skin biopsy showed epidermal atrophy and histiocytic infiltrate throughout the entire dermis, both interstitial and perivascular. The patient was diagnosed with IGDR. We decided not to change her medications because her chronic diseases were well-controlled on these medications; her skin lesion was asymptomatic, very mild, and localized to a small body area; and, lastly, IGDR is not associated with any complications in the future.

## Introduction

Interstitial granulomatous drug reaction (IGDR) is a rare drug-induced inflammatory skin condition first described by Magro et al. in 1998 [1]. Although the pathophysiology of IGDR remains unknown, the most recent theory argues that it is caused by a cell-mediated immune reaction to drugs containing unknown dermal products and/or a disruption in collagen fiber degradation, which ultimately causes interstitial granulomatous inflammation [2]. Patients with interstitial granulomatous disorders are mostly in their 40s and 50s, with a female predominance [3]. Onset typically occurs months to years after starting the offending medication. This reaction is induced by different drug classes, in particular, angiotensin-converting enzyme inhibitors, calcium channel blockers, lipid-lowering agents, antihistamines, anticonvulsants, antidepressants, diuretics, and antitumor necrosis factor agents [4]. IGDR is characterized by asymptomatic or slightly pruritic annular erythematous-to-violaceous plaques that primarily affect the intertriginous areas, lateral trunk, and extremities [5]. IGDR completely regresses after the removal of the causative agent [4]. Here, we report a case of IGDR.

## Case presentation

**Figure 1:**
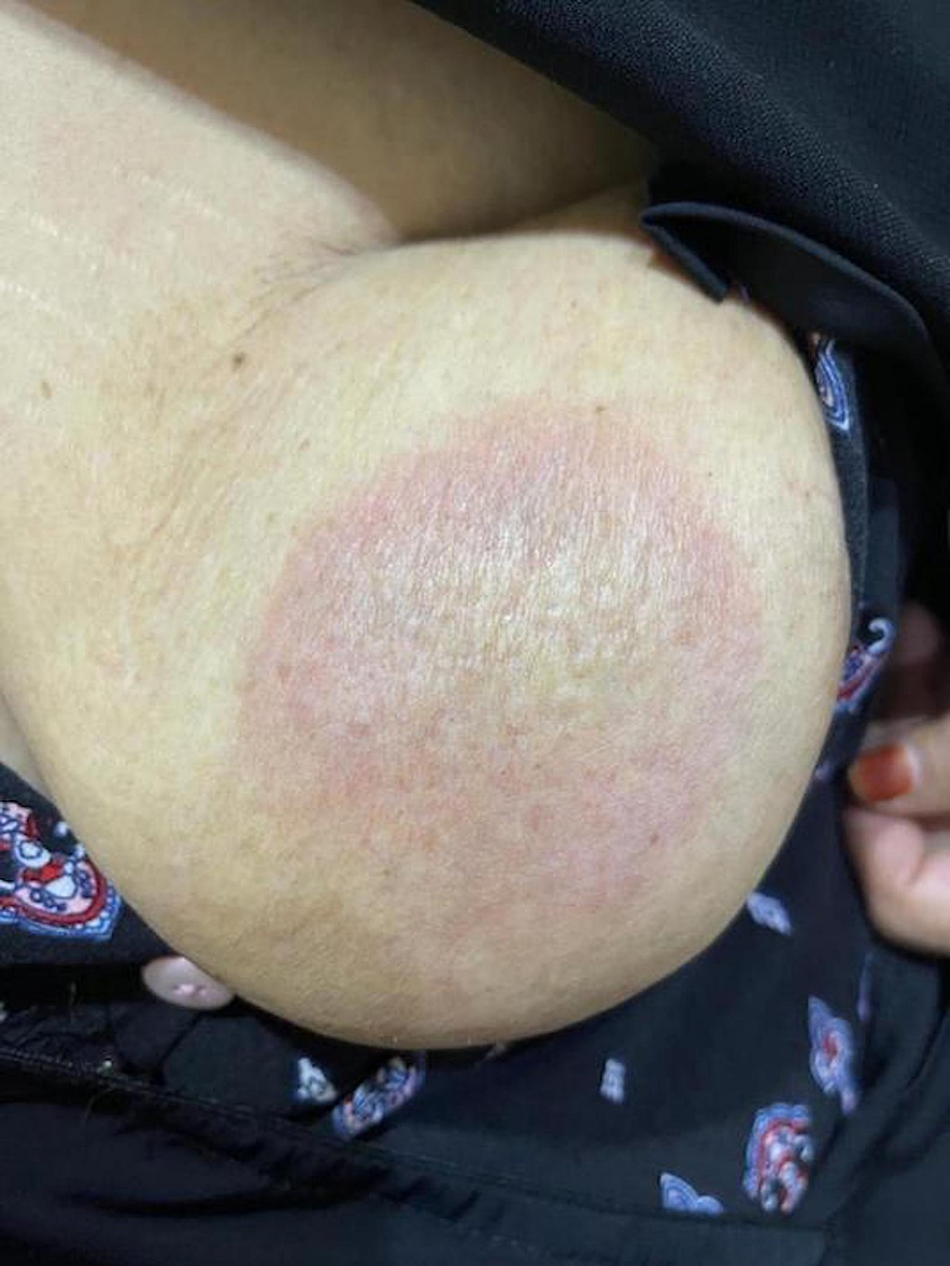
The left breast of the patient showing the non-scaly, well-demarcated, annular, erythematous patch.

**Figure 2:**
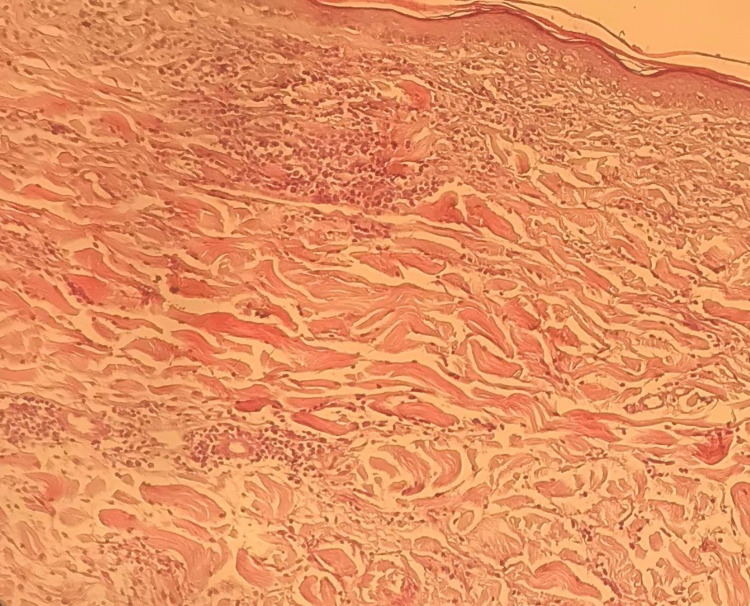
Skin biopsy showing epidermal atrophy. The dermis shows interface changes, histiocytic infiltrate, both interstitial and perivascular, throughout the dermis (hematoxylin & eosin stain; original magnification, ×20).

A 55-year-old female presented with an asymptomatic persistent skin lesion on her left breast for one year. Her medical history revealed that she had diabetes mellitus, hypertension, and dyslipidemia for several years. Her current medications included gliclazide, sitagliptin, metformin, indapamide, rosuvastatin, and valsartan. She had no history of an autoimmune disease or arthritis. A review of the systems was unremarkable. Skin examination showed a solitary 5 × 5 cm, non-scaly, well-defined, annular, erythematous patch on her left breast without induration (Figure 1). Hair, nail, and mucous membrane examination was normal. There was no regional lymphadenopathy. Histopathology of a skin biopsy specimen revealed epidermal atrophy, interface changes, and an interstitial and perivascular histiocytic dermal infiltrate (Figure 2). Laboratory tests, including complete blood cell count, liver function test, urea, creatinine, electrolytes, thyroid function test, urine analysis, antinuclear antibodies, and serology for human immunodeficiency virus and hepatitis B and C, were normal, within normal limits, or negative. The patient was seen by a breast surgeon who reported no underlying breast cancer. Her mammogram was normal. Based on the clinicopathological findings, the patient was diagnosed with IGDR. We decided not to change her medications because her chronic diseases were well-controlled by these medications; her skin lesion was asymptomatic, very mild, and localized to a small body area; and, lastly, IGDR is not associated with any complications in the future.

## Discussion

The term “reactive granulomatous dermatitis” was coined to describe a group of granulomatous inflammatory skin reactions with nearly identical clinical and histological characteristics, such as palisaded neutrophilic granulomatous dermatitis (PNGD), interstitial granulomatous dermatitis (IGD), and IGDR [6]. Clinical and histological characteristics can aid in the differentiation of these disorders (Table 1). Our patient presented with a characteristic erythematous patch with an unusual site of involvement as there have been no previous reports of IGDR localized to the breast. In our patient, the clinical differential diagnosis included patch-type granuloma annulare, the inflammatory stage of morphea, erythema migrans, lichen sclerosus et atrophicus, mycosis fungoides, IGD, PNGD, and metastasis from underlying breast cancer. The patient was seen by a breast surgeon who reported no underlying breast cancer. Her mammogram was also normal. IGDR presenting as a solitary lesion, as in our case, has been reported earlier. Other variants previously reported in the literature include erythrodermic, erythema nodosum-like lesion, and subcutaneous nodules on the palms and soles [7]. The histopathologic differential diagnoses in our case included patch-type granuloma annulare, IGD, and PNGD [8]. Our patient showed the typical histopathological findings of IGDR, including diffuse interstitial infiltrate of histiocytes and lymphocytes with vacuolar interface dermatitis, lack of neutrophils, and no collagen degeneration. However, the absence of lymphoid atypia and eosinophils points against a diagnosis of IGDR. IGD in our case could be idiopathic, drug-induced, or associated with underlying connective tissue diseases (CTDs) or autoimmune diseases. Our patient most likely had IGDR for the following reasons: she has no history of CTDs, laboratory findings were normal, ANA was negative, and she presented with most of the histopathological findings of IGDR. The diagnosis of IGDR is confirmed by the challenge and rechallenge test. However, we preferred not to do this for the following reasons: her chronic diseases were well-controlled on her medications, her skin lesion was asymptomatic, and, lastly, her skin lesion was not associated with any future complications.

**Table 1:** The differentiation between the different types of reactive granulomatous dermatitis.

	Interstitial granulomatous dermatitis	Palisaded neutrophilic granulomatous dermatitis	Interstitial granulomatous drug reaction
Associations	Rheumatoid arthritis, seronegative arthritis, and autoimmune thyroiditis [9]	Connective tissue diseases, including rheumatoid arthritis, systemic lupus erythematosus, and eosinophilic granulomatosis with polyangiitis [9]	Angiotensin-converting enzyme inhibitors, calcium channel blockers, lipid-lowering agents, antihistamines, anticonvulsants, antidepressant, diuretics, and antitumor necrosis factor agents [4]
Clinical features	Erythematous or skin-colored patches, papules, and plaques often with round, annular configuration or cord-like (rope sign), usually found on the lateral trunk, axillae, medial thighs, and buttocks [9,10]	Erythematous annular plaques or papules with central umbilication, symmetrically distributed on extensor surfaces [9]	Erythematous-to-violaceous annular plaques mainly involving the intertriginous areas, lateral trunk, and extremities [5]
Histopathology	Diffuse dermal infiltrate of lymphocytes, eosinophils, histiocytes, and focal degeneration of collagen (floating sign), without evidence of vasculitis [10]	Intense dermal infiltrate of neutrophils and interstitial histiocytes with leukocytoclastic vasculitis [9,10]	Vacuolar interface dermatitis with diffuse interstitial infiltrate of histiocytes and lymphocytes, prominently eosinophils, with or without lymphoid atypia, lack of neutrophils, and minimal collagen degeneration [2]

## Conclusions

IGDR is an uncommon inflammatory drug reaction to a wide range of drugs. A careful history and histological findings are crucial for diagnosis. More research is needed to confirm the role of these drugs in the development of IGDR.
